# Traditional medicine treatment for thoracic outlet syndrome: A protocol for systematic review of randomized controlled trials

**DOI:** 10.1097/MD.0000000000021074

**Published:** 2020-07-02

**Authors:** Ji Hye Hwang, Sujeong Ku, Jin-Ho Jeong

**Affiliations:** a Department of Acupuncture and Moxibustion Medicine, College of Korean Medicine, Gachon University, Seongnam; b Department of Clinical Korean Medicine, Graduate School, Kyung Hee University; c Jisung-Kyunghee Korean Medicine Clinic, Seoul, Republic of Korea.

**Keywords:** acupuncture, protocol, systematic review, traditional Chinese medicine, thoracic outlet syndrome, traditional Korean medicine

## Abstract

**Background:**

Diagnosis of thoracic outlet syndrome (TOS) is challenging; however, proper evaluation and treatment ensure relief from symptoms in most patients. A comprehensive approach to treatment is important, considering the multifactorial etiology of TOS. The objective of this systematic review is to describe the methods for evaluating the effectiveness and safety of acupuncture-based traditional medicine treatments for TOS.

**Methods:**

A total of 13 databases will be searched, from their inception to the present date, for studies that have investigated the treatment of TOS. Databases that will be included are MEDLINE, Embase, AMED, Cochrane Library, CINAHL, and 4 Korean, 2 Chinese, and 2 Japanese databases.

We will include randomized controlled trials (RCTs) assessing acupuncture-based traditional medicine for the treatment of any type of TOS. All RCTs on traditional medicine with any form of acupuncture will be eligible for inclusion. The methodologic quality of the RCTs will be analyzed using the Cochrane Collaboration tool to assess the risk of bias, and the confidence in the cumulative evidence will be assessed using the grading of recommendations assessment, development, and evaluation instrument.

**Ethics and dissemination:**

The results of this systematic review will be published in a peer-reviewed journal and disseminated both electronically and in print. The review will be updated to inform and guide health care practices.

**Trial registration number:**

PROSPERO 2020 CRD42020164869

## 1 Introduction

Thoracic outlet syndrome (TOS) was first described by Peet et al in 1956.^[1]^ TOS is defined as a disorder that causes pain in the upper extremities, shoulder, and neck by compressing the neurovascular bundle exiting the thoracic outlet, which is constrained by the anterior middle scalene muscles, clavicle, and first rib.^[2–5]^ TOS should be distinguished from other pain-causing disorders of the neck and shoulder area, such as tears of the rotator cuff, cervical spondylosis, supraspinatus tendonitis, and adhesive capsulitis, among others.^[6]^

The TOS is classified into subgroups based on the pathophysiology as neurogenic (nTOS), venous (vTOS), and arterial (aTOS).^[2]^ Each TOS subgroup could be related to congenital causes such as the existence of a cervical rib or atypical first ribs, traumatic causes such as whiplash injuries and falls, or functionally acquired causes such as active and repetitive activities related to sports or work.^[7]^

Diagnosis of TOS generally relies on the clinical familiarity of the condition with assessment of symptoms and patient-specific risk factors, and can be confirmed by provocative physical examination maneuvers, radiographic, and/or vascular studies. Although it has been reported that it is difficult to estimate the actual incidence of TOS due to the extensive etiology and lack of expert consensus on diagnostic tests, several previous studies have reported an incidence of 3 to 80/1000 individuals.^[7]^ It has also been reported that nTOS occurs most commonly, representing approximately 95% of the total cases, whereas vTOS and aTOS represent approximately 3% to 5% and 1% to 2% of the cases, respectively.^[8]^ Symptoms of TOS appear in individuals between 20 and 50 years of age, and are more common in women.^[9]^

Due to the multifactorial etiology, the best treatment for TOS includes a comprehensive and multidisciplinary approach, with surgery, lifestyle modifications, pain management, anticoagulation, physical therapy, and rehabilitation.^[10]^ Management strategies depend on the underlying etiology of the condition. Initial treatment of nTOS comprises conservative measures, whereas patients with vTOS or nTOS with refractory symptoms may undergo surgical management.^[11]^ However, a multimodal treatment approach including patient education, TOS-specific rehabilitation, and pharmacologic therapies have shown positive results.^[11]^ Pharmacologic interventions often provide symptomatic relief, and primarily include analgesics (nonsteroidal anti-inflammatory drugs and/or opioids) for neuropathic pain, as well as muscle relaxants, anticonvulsants, and/or antidepressants as adjuvant.^[10]^ Surgery for TOS is indicated in patients who do not respond to conservative management. The threshold for decompression varies widely for mild to moderate symptoms; however, certain symptoms require surgery.^[3]^ Physical therapy and conservative management of nTOS should be continued for at least 4 to 6 months prior to consideration of surgical intervention.^[12]^ However, surgery is frequently indicated as the initial intervention in patients with arterial or venous TOS.^[3]^

The best standard primary treatment for TOS has not been established, despite the presence of several conservative options. Traditional East Asian medicine including traditional Korean medicine (TKM) and traditional Chinese medicine (TCM) has been used as one of the nonsurgical treatment options for TOS in clinical practice; however, there are limited reports on the effectiveness and safety of such modalities. TKM or TCM treatment methods such as acupuncture, bee venom acupuncture, pharmacopuncture, chuna, and herbal medicine can be used in parallel; however, no standard treatment has been established. In addition, there are limited clinical studies on the effectiveness of traditional medical treatments on TOS.^[6,13]^

Acupuncture has been used for more than 3000 years as a chief component of traditional medicine and is generally considered a safe and effective remedy for pain relief and in neurologic conditions.^[14]^ Furthermore, there is moderate evidence that acupuncture can relieve chronic pain in neck disorders.^[15]^ This proposed systematic review aims to describe the methods for evaluating the evidence on the effectiveness and safety of acupuncture-based traditional medicine treatments for TOS.

## 2 Methods

### 2.1 Study registration

This systematic review adheres to the Preferred Reporting Items for Systematic Reviews and Meta-Analyses protocols (PRISMA-P).^[16]^ This protocol has been registered in PROSPERO (an international prospective register of systematic reviews) 2020, with the number/ID CRD42020164869 (https://www.crd.york.ac.uk/prospero/display_record.php?ID=CRD42020164869).

### 2.2 Ethics and dissemination

This systematic review will be published in a peer-reviewed journal and disseminated both electronically and in print. The review will be updated to inform and guide health care practices. Ethical approval is not required as this study is based on a review of published literature.

### 2.3 Data sources

Databases and search terms will be determined through discussion between all authors before the literature searches are executed. The following electronic databases will be searched for studies from their inception to inception to the present date that investigated the treatment of TOS: MEDLINE, Embase, AMED, Cochrane Library, CINAHL, 4 Korean databases (KoreaMed, OASIS, KISS, and Korean Traditional Knowledge Portal [KTCKP]), 2 Chinese databases (China National Knowledge Infrastructure [CNKI] and WanFang Data), and 2 Japanese databases (CiNii and JAIRO).

### 2.4 Criteria for inclusion of studies in this review

#### 2.4.1 Types of studies

Prospective randomized controlled trials (RCTs) that have evaluated the effectiveness of acupuncture-based treatments, such as acupuncture, electro-acupuncture, pharmacopuncture, acupotomy, bee venom acupuncture, cupping, and warm acupuncture, for TOS will be considered. Trials with any type of control intervention will be included. No language restrictions will be imposed.

#### 2.4.2 Types of participants

Patients with TOS will be included with no restrictions on other conditions, such as age, sex, country of origin, or severity of symptoms.

#### 2.4.3 Types of interventions and controls

The RCTs that have included acupuncture-based treatments as the sole treatment or as an adjunct to other treatments will be considered. The only criterion for inclusion of RCTs with other treatments is that the same treatment should have been provided to both control and intervention groups. Furthermore, trials that have compared acupuncture-based treatments with any type of control intervention will be included. Studies that have evaluated any type of acupuncture (i.e., acupuncture, electro-acupuncture, pharmacopuncture, acupotomy, bee venom acupuncture, cupping, or warm acupuncture) will be eligible for inclusion.

Other interventions included acupuncture, herbal/western medicine, cupping, chuna, diet therapy, and physical therapy, including hot pack, transcutaneous electrical nerve stimulation, interferential current therapy, ultrasound, massage, and exercise.

#### 2.4.4 Type of outcome measures

##### 2.4.4.1 Primary outcomes

Pain intensity will be analyzed using relevant confirmed pain measurement scales such as visual analog scale, verbal rating scale, and numerical rating scale, or by assessment of the quality of life.

##### 2.4.4.2 Secondary outcomes

We will analyze other scales or questionnaires that evaluate pain, functional disability, or the quality of life, the success rate of treatment (post-treatment reduction in scores by 50% compared to baseline), rates of recurrence and complications, and adverse events.

For analysis of the acupuncture-based interventions, we will summarize each item based on the types and methods, acupuncture points, extraction methods, type of syringe used, and amount, depth, and angle of the injection following the STRICTA (Standards for Reporting Interventions in Clinical Trials of Acupuncture) recommendations.

### 2.5 Data collection, extraction, and assessment

#### 2.5.1 Selection of studies

Databases and search terms will be determined through discussion between all authors before the literature searches are executed. Two reviewers (JJ and SK) will perform the electronic literature searches. Another reviewer (JHH) will resolve disagreements (if any) pertaining to study selection.

#### 2.5.2 Data extraction and quality assessment

We will review all articles retrieved through the database search to evaluate their eligibility for inclusion. In case of uncertainties, the authors will be contacted for further information. Subsequently, we will extract the following data from the selected articles: author, year of publication, study design, participants (age, sex), diseases or disorders, pharmacopuncture intervention, control intervention, outcome measures, main results, and adverse events.

**Figure 1:**
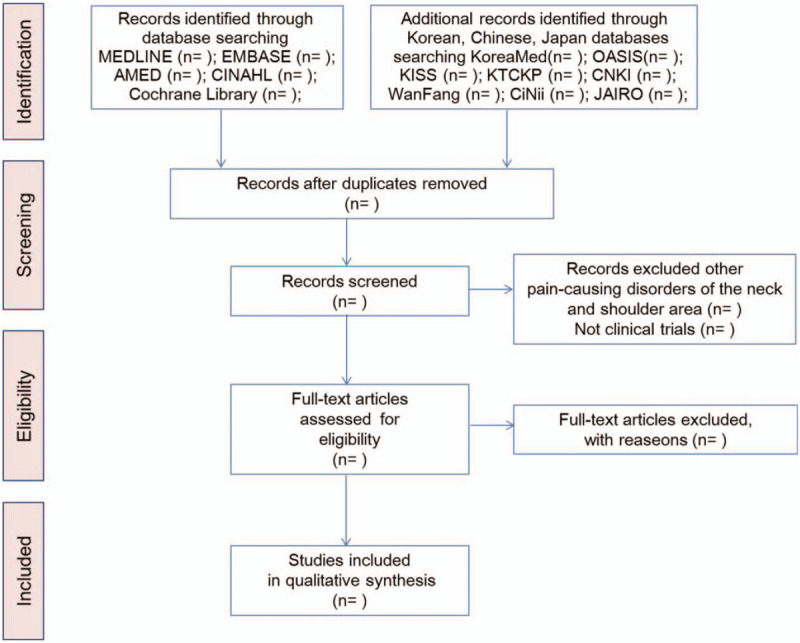
Process of the systematic review.

Two reviewers (JJ and SK) will conduct the data extraction using a recognized data extraction form accepted by all reviewers. The data will include author name(s), age, country, year of publication, characteristics of participants, intervention, method of randomization, blinding, control treatment, main outcomes, and adverse events. The reviewers will perform quality assessment using a predefined data extraction form. The flow chart of this systematic review is shown in Figure 1.

#### 2.5.3 Assessment of risk of bias

Quality assessment will be performed using the tool for assessing risk of bias from the Cochrane Handbook for Systematic Reviews of Interventions,^[17]^ which takes into account random sequence generation, allocation concealment, blinding of participants and personnel, blinding of outcome assessment, incomplete outcome data, selective reporting, and other sources of bias. The results of the assessments will be presented using the following scores: “L” indicating a low risk of bias, “U” indicating an uncertain risk of bias, and “H” indicating a high risk of bias. Any disagreement will be resolved through discussion among all authors. An arbiter (JHH) will make the final decision in cases where disagreements are not resolved by discussion.

### 2.6 Data synthesis

Differences between the intervention and control groups will be assessed. Mean differences (MDs) with 95% confidence intervals (CIs) will be used to measure the effects of treatment for continuous data. We will convert other forms of data into MDs. For outcome variables on different scales, we will use standard MDs with 95% CIs. For dichotomous data, we will present the treatment effects as relative risks with 95% CIs, and other binary data will be converted into relative risk values.

All statistical analyses will be conducted using the software program Review Manager version 5.3 (Copenhagen, The Nordic Cochrane Centre, the Cochrane Collaboration, 2014) for Windows. We will contact the corresponding authors of the studies with missing information to acquire and verify the data, whenever possible. When appropriate, we will pool the data across studies to conduct a meta-analysis using fixed or random effects. We will use GRADEpro software from Cochrane Systematic Reviews to create a Summary of Findings table.

Intention-to-treat analyses, including all randomized patients, will be performed to address missing data. A last observation carry-forward analysis will be performed in cases with missing outcome data. The original source or the published trial reports of the data will be reviewed in cases where individual patient data are unavailable initially.

Subgroup analyses will be performed to identify the possible causes in cases of heterogeneity (defined by results of tests of heterogeneity that indicate *P* < .1 via Chi-squared tests and Higgins *I*^2^ ≥ 50%).^[18]^

## 3 Discussion

The diagnosis of TOS is challenging; however, proper evaluation and treatment can ensure relief from symptoms in most patients. A comprehensive and multidisciplinary approach to the treatment is important, considering the multifactorial etiology of TOS. Traditional medicine has strengths in the treatment of conditions with multifactorial etiology.^[3]^ Acupuncture has been reported to alleviate pain and discomfort by rebalancing the energy (Qi) and blood circulation in the meridians in diseases with multifactorial etiology.^[6]^

There are limited reviews in the literature on TOS, and few mention about treatment using traditional medicine, including acupuncture. One review^[19]^ reported on the level of evidence of treatments for TOS. The review did not report any significant reduction in pain following transaxillary first rib resection compared to supraclavicular neuroplasty, and the quality of evidence of the study was low; moreover, there was no evidence that either treatment was better than no treatment. Furthermore, injection of botulinum toxin did not show significant improvement compared to placebo saline injections, and the level of evidence of the study was considered moderate. It also reported that there are no RCTs of other treatments currently used for TOS.

In 2018, a review was published on TKM, which reported that a conservative approach was the priority for the treatment of TOS. The review suggested that the TKM methods such as acupuncture, acupotomy, pharmacopuncture, and chuna therapy had positive effects on patients with TOS.^[13]^ However, this study included some case reports and systematic review articles and did not follow the formal recommendations for a systematic review.

Therefore, the authors of this study would like to systematically review the impact of TKM and TCM on the treatment of TOS. Evidence of the safety and effectiveness of traditional medicine treatments for TOS will be assessed in this systematic review. The results of this study can be used by clinicians to manage patients with symptoms of TOS.

The evidence obtained from this systematic review will provide useful information to patients, practitioners, and health policy makers. Furthermore, patients suffering from TOS will be able to receive appropriate traditional medicine treatments, while doctors will be able to confirm the basis for treatment decisions. From a policy viewpoint, outcomes of this study are also expected to provide basic information to determine the health insurance coverage for traditional medicine treatments and may be used as evidence to establish an integrated model of East-West treatment for TOS.

## Author contributions

JHH and JJ conceived this study and developed the criteria; SK and JJ performed the selection of studies for inclusion in the review. JHH wrote this protocol and revised this manuscript. All authors have read and approved this manuscript.
